# Detailed Ophthalmic and Pathological Features of Choroidal Metastasis From Breast Cancer: A Case Series of Five Patients

**DOI:** 10.7759/cureus.83484

**Published:** 2025-05-04

**Authors:** Toshihiko Matsuo, Takehiro Tanaka, Tadahiko Shien, Atsushi Muraoka, Hiroyoshi Doihara

**Affiliations:** 1 Division of Healthcare Science, Graduate School of Interdisciplinary Science and Engineering in Health Systems, Okayama University, Okayama, JPN; 2 Department of Ophthalmology, Okayama University Hospital, Okayama, JPN; 3 Department of Pathology, Graduate School of Medicine, Dentistry, and Pharmaceutical Sciences, Okayama University, Okayama, JPN; 4 Department of Breast and Endocrine Surgery, Okayama University Hospital, Okayama, JPN; 5 Department of Surgery, Kagawa Rosai Hospital, Marugame, JPN; 6 Department of Surgery, General Medical Center, Kawasaki Medical School, Okayama, JPN

**Keywords:** breast cancer, chemotherapy, choroidal metastasis, estrogen receptor, her2, immunostaining, invasive ductal carcinoma, ki-67, progesterone receptor, radiation

## Abstract

Breast cancer causes choroidal metastases on rare occasions. This study presented the eye manifestations of choroidal metastases from breast cancer and their response to treatments in detail as well as their pathological correlation in five patients. The patients' age at the diagnosis of breast cancer ranged from 24 to 69 years (median: 37 years). The time from the diagnosis of breast cancer to the detection of metastases was concurrent in one patient, two years later in three patients, and six years later in the other patient. The time from the detection of systemic metastases to the detection of choroidal metastases was the same in one patient, while it ranged from one to seven years later in four patients. Choroidal metastases were in the unilateral eye of four patients, whereas they were in both eyes of one patient. Choroidal metastases manifested as one or a few nodular or flat choroidal lesions with serous retinal detachment. As for the treatment of choroidal metastases, enucleation of the right eye was chosen based on the patient's wish as well as the family's wish in the earliest patient when cancer notification was not the norm in Japan. In the other four patients, whole-eye radiation was performed to reduce the choroidal metastatic lesions. As regards the prognosis, which was available in four patients, three patients died within one year from the diagnosis of choroidal metastases, while one patient died one year and eight months later. Regarding the pathology of breast cancer, which was available in four patients, immunostaining of the preserved enucleated eye in the earliest patient revealed that breast cancer cells in the choroidal metastatic lesion were positive for estrogen receptor and negative for progesterone receptor and human epidermal growth factor receptor 2 (HER2). Invasive ductal carcinoma in two patients was positive for estrogen receptor and negative for HER2, while invasive ductal carcinoma in the other patient was triple-negative for estrogen receptor, progesterone receptor, and HER2 with a high Ki-67 index. In conclusion, the prognosis for life was poor in patients with breast cancer who developed choroidal metastases. Choroidal metastatic lesions showed a response to whole-eye radiation to improve the quality of vision at the end of life. Vision-related symptoms should be monitored in the course of chemotherapy for systemic metastases.

## Introduction

Choroidal nodular lesions, often in association with serous retinal detachment, are manifestations of choroidal tumors, which are extremely rare in ophthalmic practice. In the case of choroidal tumors, primary tumors and metastatic choroidal lesions should be considered first in the differential diagnosis to determine therapeutic options. Choroidal malignant melanoma is the most frequent entity in primary choroidal tumors [1,2]. Metastatic choroidal tumors would follow next in the incidence [3-5]. Among metastatic choroidal tumors, metastases from lung cancers and breast cancers are well-recognized in a large series of patients [3,4].

In this study, we reviewed five patients with breast cancer who developed choroidal metastases in the period of 36 years from 1987 to 2023 at a single referral-based institution. Currently, the treatment strategy for breast cancer is stratified according to the immunostaining data of cancer cells: whether they express hormone receptors such as estrogen and progesterone receptors as well as human epidermal growth factor receptor 2 (HER2), which is a receptor tyrosine-protein kinase erbB-2 [6-8]. Furthermore, genetic variants in germline *BRCA1* and *BRCA2* and other genes are examined to determine therapeutic options [7]. In the era of continuing advances in the therapeutic options for patients with breast cancer metastases, their long-term survival would be expected, and thus, their quality of life, as well as their quality of vision, would be a crucial point to be considered in the standard of care. We also tried in this study to summarize the immunostaining patterns of breast cancer in the five present patients with choroidal metastases.

## Case presentation

Case 1

**Figure 1:**
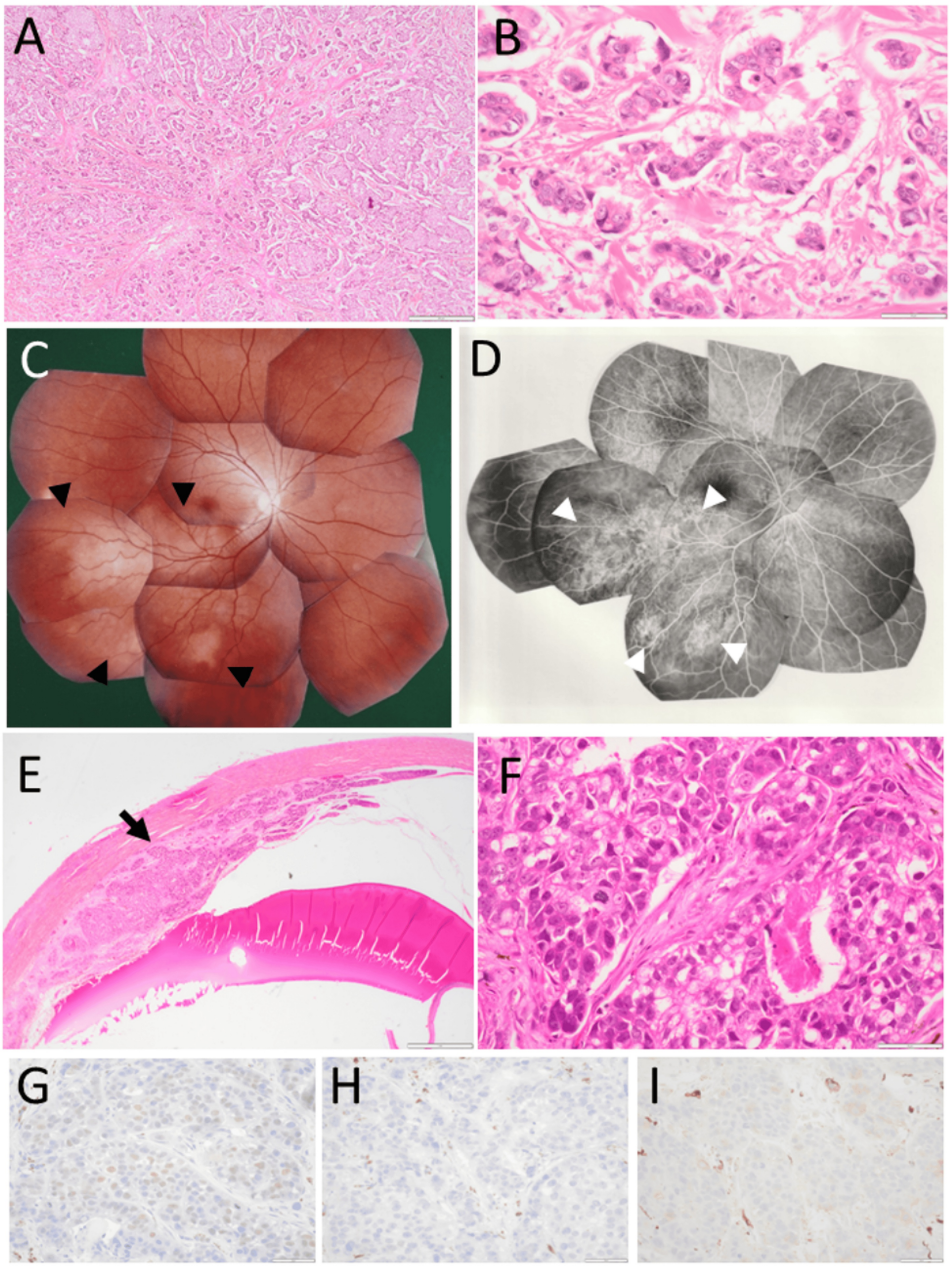
Case 1: pathology and fundus photographs Left breast cancer at the age of 32 years, showing invasive ductal carcinoma (A, B) in hematoxylin-eosin stain. Montaged fundus photographs (C) and fluorescein angiograms (D) of multiple choroidal metastatic lesions (arrowheads) in the right eye at the age of 35 years. Immunostaining of sections of the paraffin-embedded half-cut eyeball which was preserved for 38 years, showing breast cancer cells in choroidal metastasis (E, F) along the sclera (arrow) positive for estrogen receptor (G) although faint and negative for progesterone receptor (H) and HER2 (I). The detached retina is not found, and the location of the lens is due to an artifact in E. Scale bar is 500 µm in A, 50 µm in B, F, G, H, and I, and 1000 µm in E. HER2: human epidermal growth factor receptor 2

A 32-year-old woman underwent total mastectomy and lymph node dissection for left breast cancer (Figure 1A, 1B) at a hospital. She was then reported to have taken tamoxifen and cyclophosphamide for bilateral lung and bone metastases. Three years later, at the age of 35 years, she noticed blurring of the superonasal quadrant of the visual field in her right eye and was referred to a university hospital. She did not have a family history of cancer or any other past history. The best-corrected visual acuity in decimals was 1.2 in the right eye and 1.5 in the left eye. The right eye had a large nodular choroidal lesion along the lower vascular arcade and two small nodules in the inferotemporal midperiphery of the fundus with serous retinal detachment (Figure 1C, 1D). The left eye was normal. Gallium-67 scintigraphy showed high uptakes in the vertebral bones, ribs, and bilateral lung fields. Due to the rapid growth of the choroidal mass, she chose enucleation of the right eye. She moved to her hometown and was lost to follow-up. Immunostaining of sections of the paraffin-embedded half-cut eyeball, which was preserved for 38 years, demonstrated that breast cancer cells in choroidal metastasis (Figure 1E, 1F) were positive for estrogen receptor (Figure 1G) and negative for progesterone receptor (Figure 1H) and HER2 (Figure 1I).

Case 2

**Figure 2:**
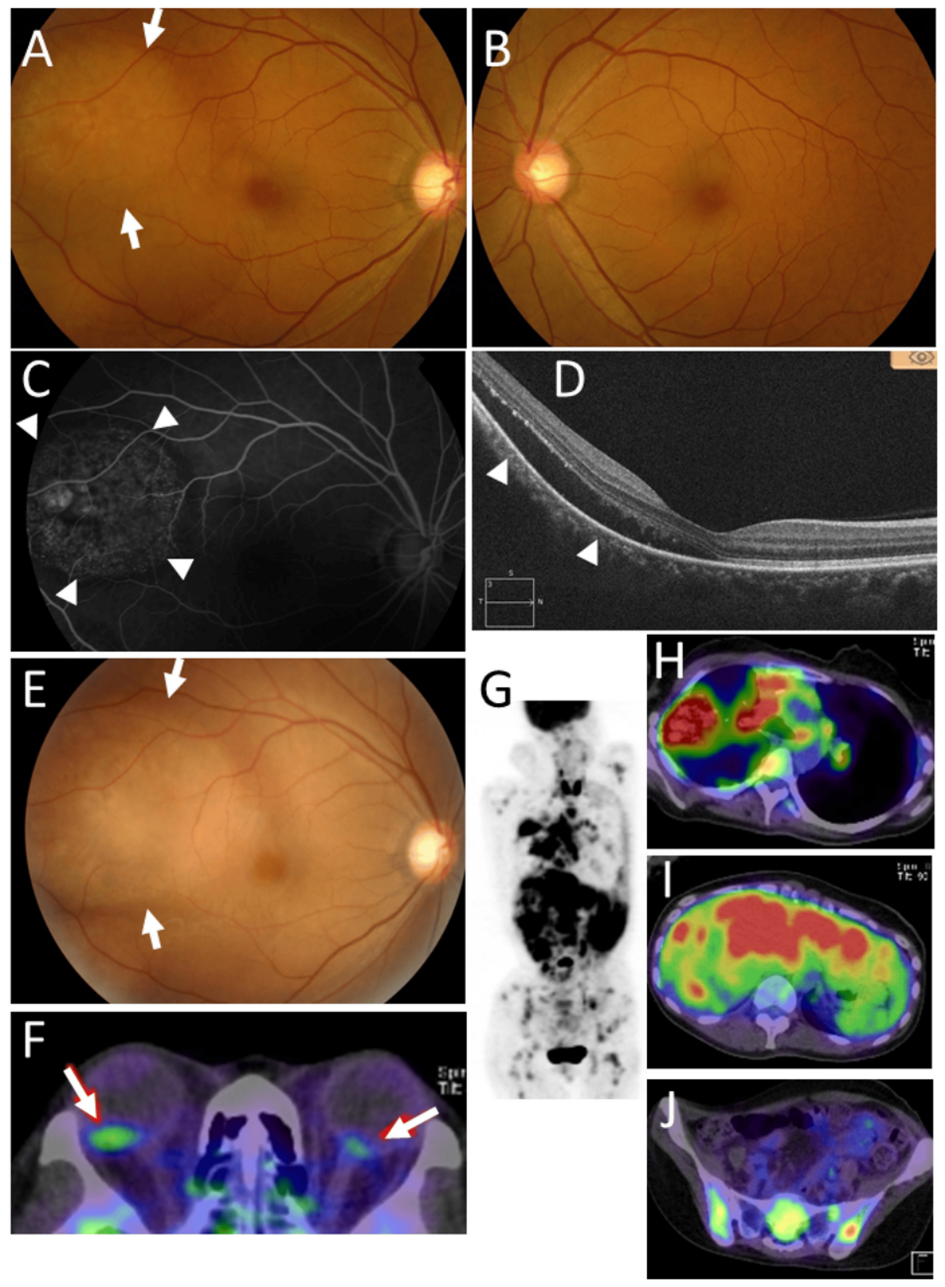
Case 2: fundus photographs, optical coherence tomography, and positron emission tomography Fundus photograph (arrows, A), fluorescein angiogram (arrowheads, C), and horizontal section of optical coherence tomography (serous retinal detachment with arrowheads, D) of choroidal metastatic lesion in the right eye at the age of 47 years, eight years later from the diagnosis of right breast cancer. Fundus photograph in the left eye with no lesion (B). Enlarging choroidal lesion (arrows, E) in the right eye one month later. Fluorodeoxyglucose positron emission tomography at this time, showing multiple uptake sites (G), indicative of metastases in the choroid of both eyes (arrows, F), lung (H), liver (I), and bones (J).

A 39-year-old woman had a right mastectomy for breast cancer and took tamoxifen. Two years later, she developed metastases in the bilateral lungs, liver, bones, and lymph nodes and underwent chemotherapy with capecitabine and cyclophosphamide. She had no other past history or family history of cancer. At the age of 47 years, she noticed blurred vision in her right eye and was referred to an ophthalmologist. The best-corrected visual acuity in decimals was 1.0 in the right eye and 1.5 in the left eye. The right eye showed one nodular choroidal lesion with serous retinal detachment in the temporal area of the fundus near the macular area (Figure 2A, 2C, 2D), while the left eye was normal (Figure 2B). In a month, the choroidal lesion enlarged rapidly to involve the macular area in the right eye (Figure 2E). Fluorodeoxyglucose positron emission tomography detected high uptakes in the posterior pole of the right eyeball (Figure 2F), bilateral lungs, liver, vertebral bones, and other bones (Figure 2G, 2H, 2I, 2J). She underwent right whole-eye radiation in a total dose of 30 Gy but died of acute liver failure in a few months.

Case 3

**Figure 3:**
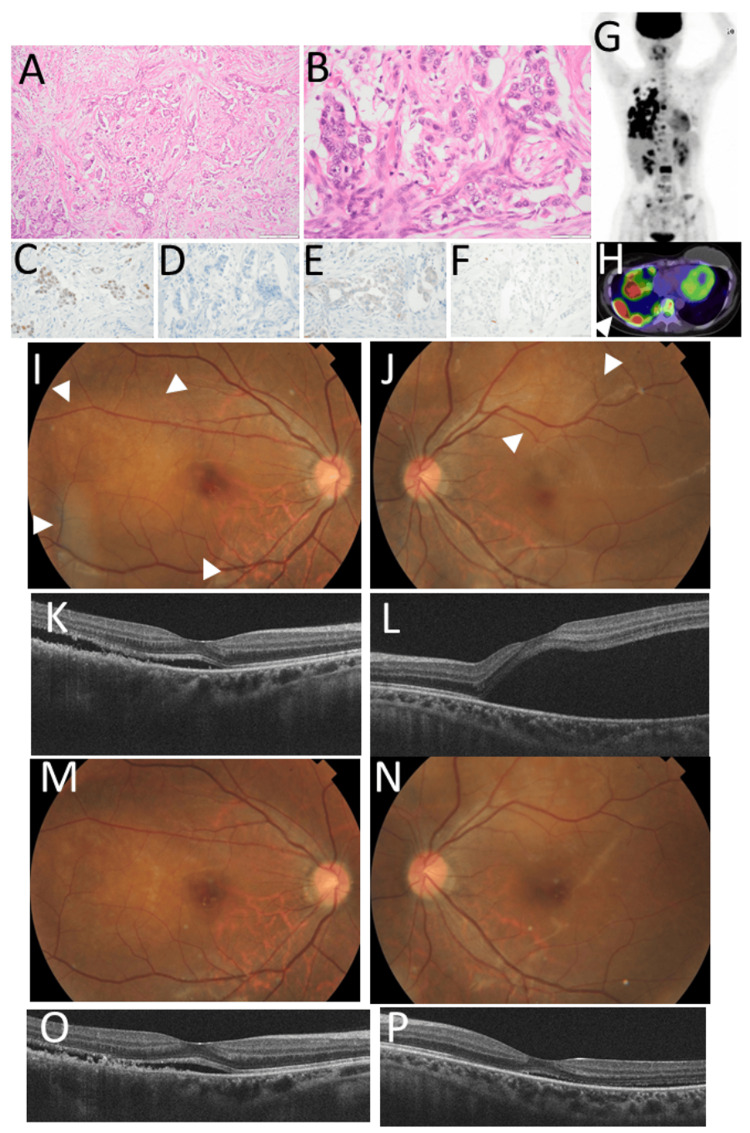
Case 3: pathology, fundus photographs, and optical coherence tomography Left breast cancer at the age of 24 years, showing invasive ductal carcinoma (A, B) in hematoxylin-eosin stain, positive for estrogen receptor (C) and negative for progesterone receptor (D) and HER2 (E). Ki-67-positive cells are minimal (F). Fluorodeoxyglucose positron emission tomography at the age of 27 years, showing multiple uptake sites (G), indicative of metastases in the lung with pleural dissemination (arrowhead, H) and bones. Fundus photographs and horizontal sections of optical coherence tomography two months later, showing choroidal metastases in both eyes (arrowheads, I: right eye, J: left eye) with serous retinal detachment (K: right eye, L: left eye). Choroidal metastatic lesions (M: right eye, N: left eye) have been reduced in size with decreased volume of serous retinal detachment (O: right eye, P: left eye) after 30 Gy whole-eye radiation. Scale bar is 200 µm in A and 50 µm in B, C, D, E, and F. HER2: human epidermal growth factor receptor 2

A 24-year-old woman underwent mastectomy and lymph node dissection with a breast implant for left breast cancer. She had no other past history or family history of cancer. The pathological diagnosis was invasive ductal carcinoma (Figure 3A, 3B), which was positive for estrogen receptor (Figure 3C) and negative for progesterone receptor (Figure 3D) and HER2 (Figure 3E). The number of Ki-67-positive cells was low (Figure 3F). She had taken tamoxifen and leuprorelin. Two years later, she developed metastases in the bilateral lungs, mediastinal lymph nodes, and multiple bones with pleural dissemination (Figure 3G, 3H) and then developed liver and brain metastases. She underwent a series of chemotherapy with different regimens: paclitaxel; fluorouracil, epirubicin, and cyclophosphamide (FEC); epirubicin and cyclophosphamide; and capecitabine and cyclophosphamide. In a year, at the age of 27 years, she noticed metamorphopsia in both eyes and was referred to an ophthalmologist. The best-corrected visual acuity in decimals was 0.9 in the right eye and 0.3 in the left eye. She had a few nodular choroidal lesions in the posterior pole of both eyes with serous retinal detachment (Figure 3I, 3J, 3K, 3L). At this time, she also had multiple brain metastatic lesions. She underwent whole-brain and bilateral whole-eye radiation at 30 Gy (3 Gy each with 10 fractions). The choroidal mass lesions diminished in both eyes (Figure 3M, 3N, 3O, 3P), and the best-corrected visual acuity was 1.2 in both eyes. She died several months later.

Case 4

**Figure 4:**
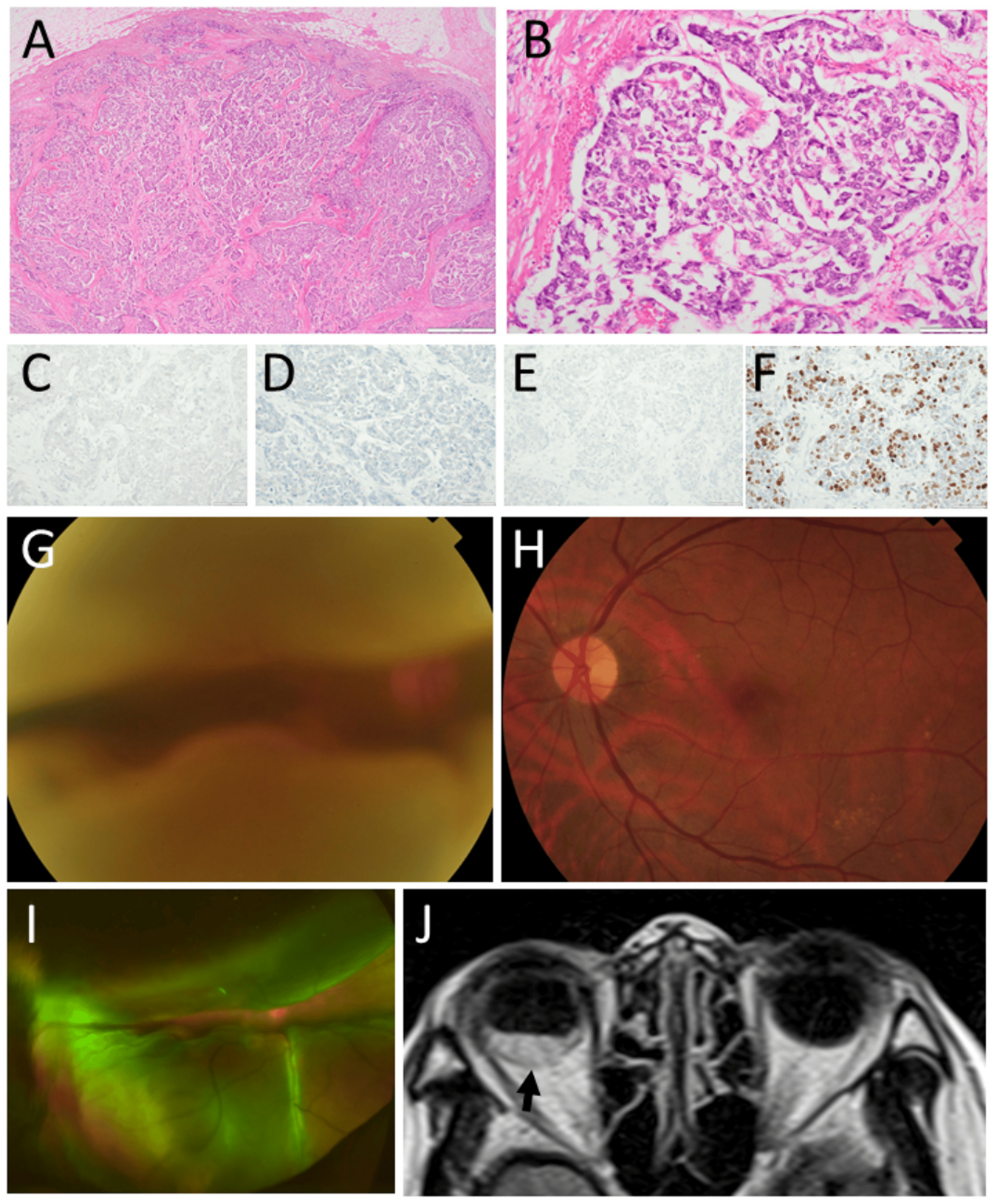
Case 4: pathology, fundus photographs, and magnetic resonance imaging Right breast cancer at the age of 69 years, showing invasive ductal carcinoma (A, B) in hematoxylin-eosin stain, triple-negative for estrogen receptor (C), progesterone receptor (D), and HER2 (E). The number of Ki-67-positive cells is high (F). Fundus photograph (G) and wide-field fundus photograph (I), showing two large choroidal masses with almost total retinal detachment in the right eye two years later. The left eye was normal (H). Magnetic resonance imaging, showing mass lesion in the posterior part of the right eye (arrow, J). Scale bar is 1000 µm in A and 100 µm in B, C, D, E, and F. HER2: human epidermal growth factor receptor 2

A 69-year-old woman underwent mastectomy and lymph node dissection for right breast cancer after a half-year period of preoperative chemotherapy with four courses of the FEC regimen and then with four courses of gemcitabine and paclitaxel. She had no other past history or family history of cancer. In pathological examinations, invasive ductal carcinoma (Figure 4A, 4B) was negative for estrogen receptor (Figure 4C), progesterone receptor (Figure 4D), and HER2 (Figure 4E), so-called triple-negative. The rate of Ki-67-positive cells (Ki-67 index) was high (Figure 4F). She was stable for one year with the oral administration of tegafur-gimeracil-oteracil combination. At the age of 71 years, she developed parasternal and mediastinal lymph node metastases and underwent mediastinal radiation at 70 Gy and chemotherapy with doxifluidine and cyclophosphamide. In the course of chemotherapy, she noticed darkness in her right eye and was referred to an ophthalmologist. The best-corrected visual acuity in decimals was hand movement in the right eye and 1.5 in the left eye. She had intraocular lens implantation in both eyes. The right eye had two large choroidal masses with almost total retinal detachment (Figure 4G, 4I, 4J), while the left eye was normal (Figure 4H). She underwent whole-eye radiation at 40 Gy (2 Gy each with 20 fractions) followed by limited-field radiation at 10 Gy (2 Gy each with five fractions) and additional boost to the remaining tumor at 6 Gy (2 Gy each with three fractions). She kept faint vision in the right eye and died of pulmonary effusion and failure five months later from the choroidal metastases.

Case 5

**Figure 5:**
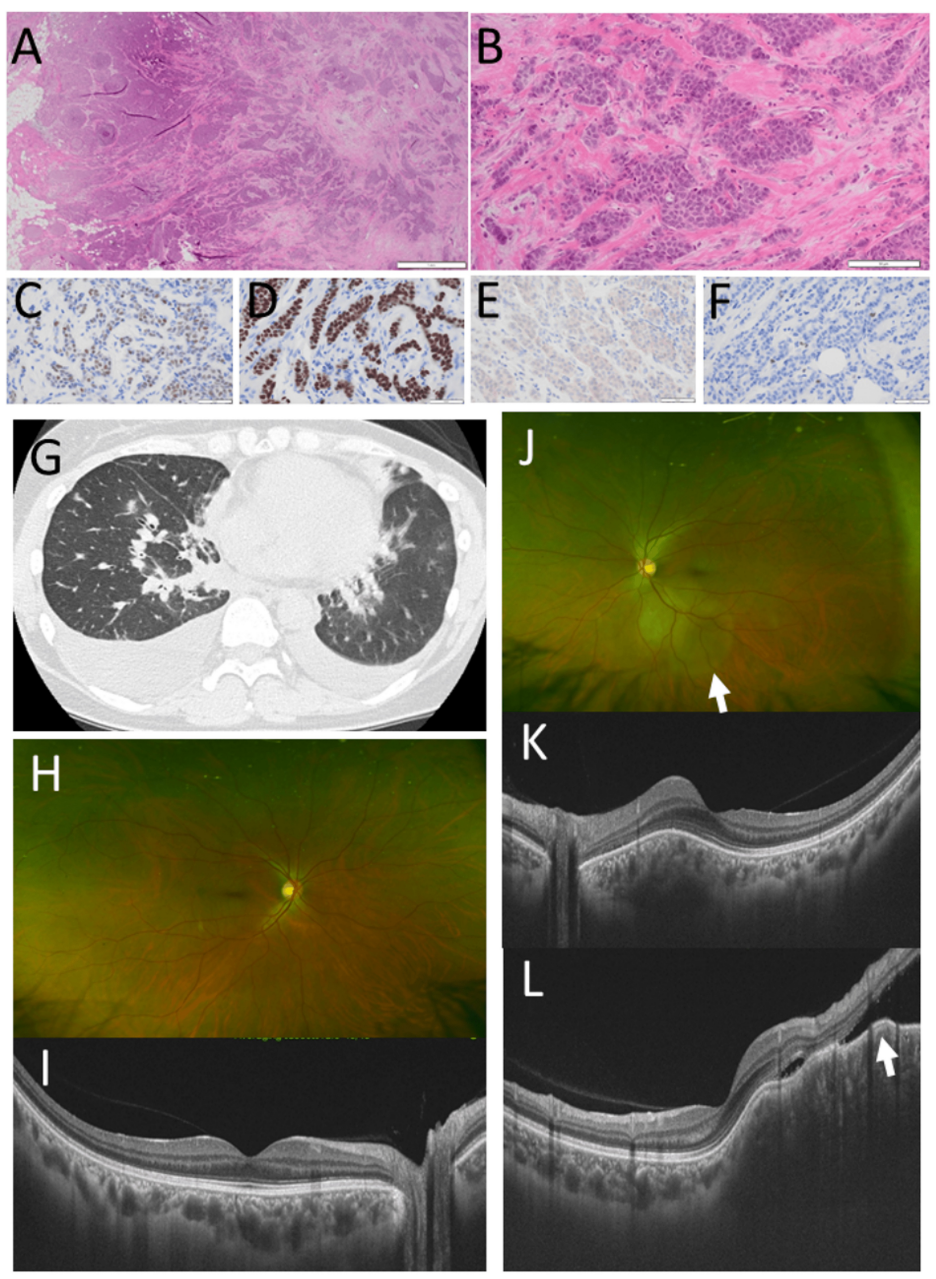
Case 5: pathology, computed tomography scan, fundus photographs, and optical coherence tomography during the initial visit to an ophthalmologist Left breast cancer at the age of 37 years, showing invasive ductal carcinoma (A, B) in hematoxylin-eosin stain, positive for estrogen receptor (C) and progesterone receptor (D) and negative for HER2 (E). The number of Ki-67-positive cells is small (F). Computed tomography scan (G), showing multiple lung lesions at the age of 50 years when she developed choroidal metastasis. Wide-field fundus photographs (H: right eye, J: left eye) and horizontal sections (I, K) and vertical section (L) of optical coherence tomography (I: right eye, K, L: left eye), showing normal fundus in the right eye (H, I) and one choroidal lesion (arrow, J) in the left eye with serous retinal detachment in the lower part (arrow, L). Scale bar is 1000 µm in A and 50 µm in B, C, D, E, and F. HER2: human epidermal growth factor receptor 2

**Figure 6:**
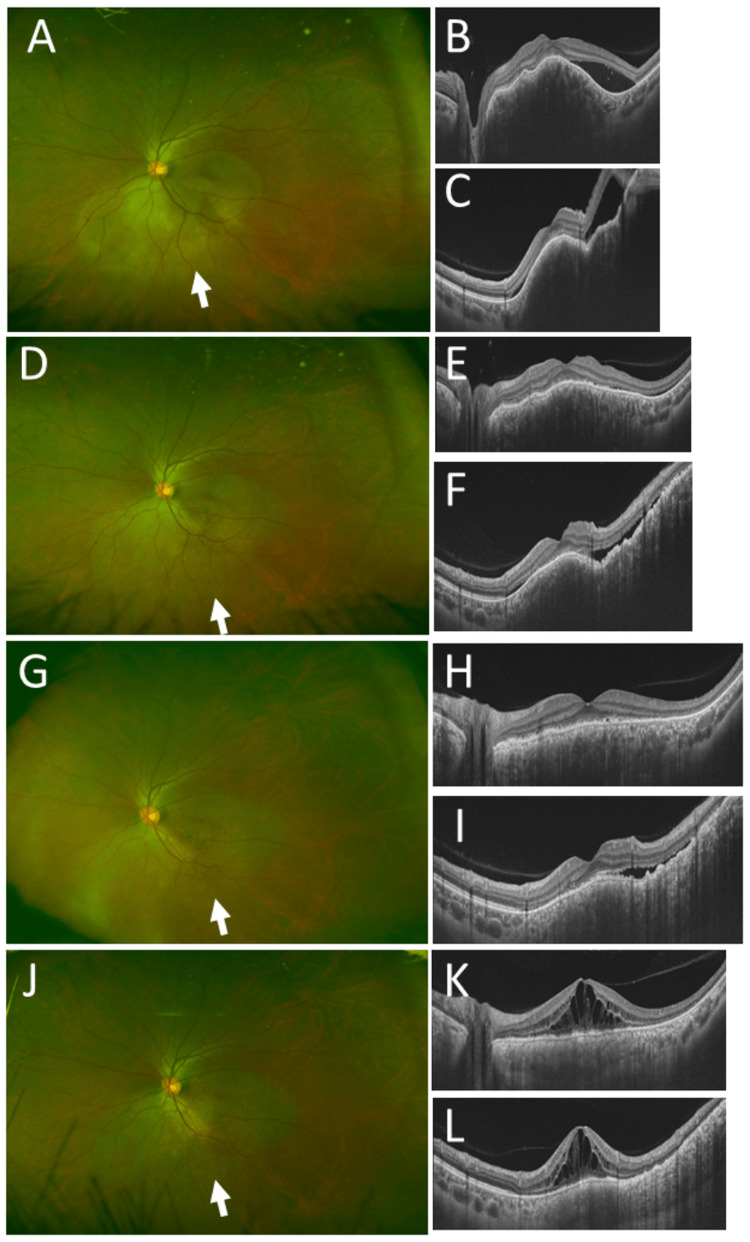
Case 5: fundus photographs and optical coherence tomography in the course of chemotherapy Sets of wide-field fundus photograph (left panel) and horizontal (top right panel) and vertical (bottom right panel) sections of optical coherence tomography in the left eye in the time sequence of the AC regimen in three months and docetaxel in six months. Choroidal lesion (arrow, A) at the beginning of the AC regimen (A, B, C) becomes smaller in one month (arrow, D, E, F), almost flattened in three months (arrow, G, H, I), and stable with macular edema (K, L) in five months (J, K, L). AC regimen: doxorubicin and cyclophosphamide

**Figure 7:**
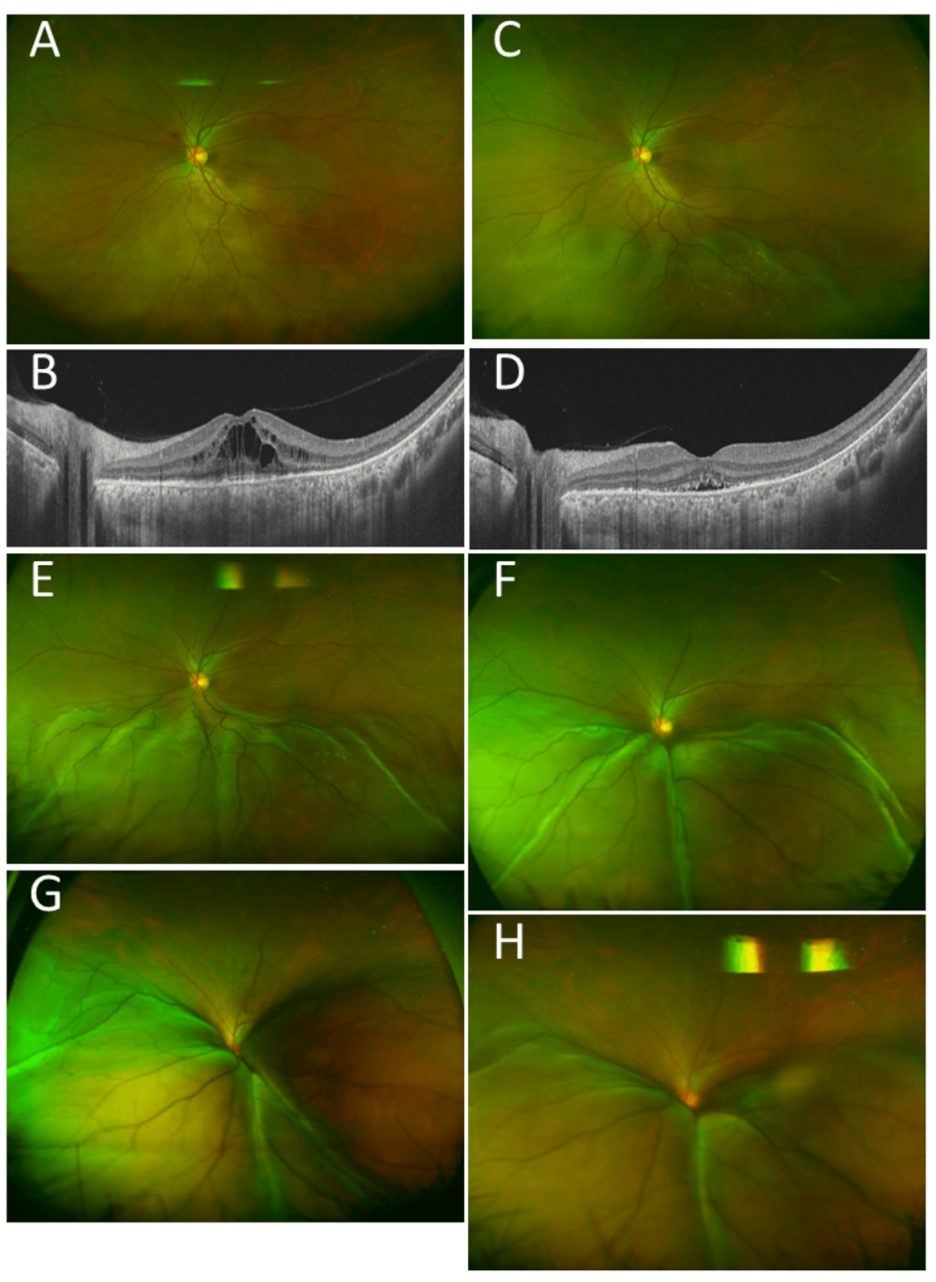
Case 5: fundus photographs and optical coherence tomography of the regrowth of choroidal metastasis Wide-field fundus photograph and horizontal section of optical coherence tomography in eight months from the start point of the AC regimen, showing stable choroidal lesion (A) and macular edema (B) in the left eye. Enlarging choroidal lesion (C) in the lower part of the left eye with no macular edema (D) in 10 months from the start point of the AC regimen when docetaxel was switched to capecitabine. Further enlarging choroidal lesion in the left eye in 11 months (E), 12 months (F), and 13 months (G) from the start point of the AC regimen. The choroidal mass became smaller with the optic disc visible in 14 months from the start part of the AC regimen, two months after the completion of 30 Gy whole-eye radiation. AC regimen: doxorubicin and cyclophosphamide

A 37-year-old woman underwent mastectomy and sentinel lymph node dissection for left breast cancer. She had no other past history and had a family history of gastric cancer in her father. In the pathological examination, invasive ductal carcinoma (Figure 5A, 5B) was positive for estrogen receptor (Figure 5C) and progesterone receptor (Figure 5D) and negative for HER2 (Figure 5E). The rate of Ki-67-positive cells was low (Figure 5F). She was stable with tamoxifen for six years until the age of 43 years when multiple metastatic lesions were detected in the bilateral lungs, bones, and mediastinal, abdominal, and paraaortic lymph nodes by fluorodeoxyglucose positron emission tomography. She underwent a series of chemotherapy with different regimens: leuprorelin and anastrozole, doxifluidine and cyclophosphamide, toremifene, fulvestrant, letrozole, and exemestane. At the age of 50 years, she noticed a round shadow in the vision of her left eye. At this time, she had multiple metastatic lesions in both lungs (Figure 5G). The best-corrected visual acuity in decimals was 1.2 in both eyes. The right eye was normal (Figure 5H, 5I), while the left eye had a nodular choroidal lesion along the lower vascular arcade of the fundus with serous retinal detachment (Figure 5J, 5K, 5L). She had chemotherapy with four courses of the AC regimen (doxorubicin and cyclophosphamide) in three months, and the choroidal metastatic lesion in the left eye became smaller (Figure 6A-6L). She then had six courses of docetaxel in six months, which was followed by capecitabine. She was revealed to have a variant of unknown significance in germline *BRCA1*. One year later from the choroidal metastasis, magnetic resonance imaging showed multiple brain metastatic lesions, and she underwent CyberKnife radiotherapy for the brain metastatic lesions. At that time, the choroidal metastatic lesion in the left eye enlarged rapidly with serous retinal detachment, and the visual acuity in the left eye dropped to hand movement (Figure 7A-7H). She decided to undergo whole-eye radiation at 30 Gy (3 Gy each with 10 fractions). She had eribulin and died seven months later, one year and eight months from the diagnosis of choroidal metastasis.

## Discussion

The clinical features of the five patients are summarized in Table 1. The patients' age at the diagnosis of breast cancer ranged from 24 to 69 years (median: 37 years). Breast cancer was on the right side in two patients and on the left side in three patients. The time from the diagnosis of breast cancer to the detection of metastases was concurrent in one patient (Case 1), two years later in three patients, and six years later in the other patient (Case 5). The time from the detection of systemic metastases to the detection of choroidal metastases was concurrent with systemic metastases in one patient (Case 4) and ranged from one to seven years later in four patients. Choroidal metastases were in the right eye of three patients, in the left eye of one patient (Case 5), and in both eyes of another patient (Case 3). The manifestations of choroidal metastases were one or a few nodular or flat choroidal lesions with serous retinal detachment. As for the treatment of choroidal metastases, enucleation of the right eye was chosen based on the patient's wish as well as the family's wish in the earliest patient (Case 1) when cancer notification was not the norm in Japan. In the other four patients, whole-eye radiation was done to reduce the choroidal metastatic lesions. As regards the prognosis, three patients died within one year from the diagnosis of choroidal metastases, while one patient died one year and eight months later (Case 5). One patient (Case 1) was lost to follow-up.

**Table 1:** Clinical features in five patients with choroidal metastasis from breast cancer Anastrozole, letrozole, and exemestane are aromatase inhibitors. Leuprorelin is an LH-RH agonist. Fulvestrant is a selective estrogen receptor downregulator. Tamoxifen and toremifene are anti-estrogen drugs. Eribulin is a tubulin inhibitor. FEC regimen: fluorouracil, epirubicin, and cyclophosphamide; AC regimen: doxorubicin and cyclophosphamide; LH-RH: luteinizing hormone-releasing hormone

Case no.	Age at the diagnosis of breast cancer	Laterality of breast cancer	Initial treatment	Age at the detection of metastasis	Metastatic sites	Additional treatment	Age at choroidal metastasis	Laterality of choroidal metastasis	Eye symptoms; manifestations; treatment	Age at death
1	32 years	Left	Mastectomy; lymph node dissection; tamoxifen; cyclophosphamide	32 years	Bilateral lungs and bones	Not known	35 years	Right eye	Blur in right-eye visual field; three flat-shaped choroidal nodules with serous retinal detachment; right-eye enucleation	Not known
2	39 years	Right	Mastectomy; tamoxifen	41 years	Bilateral lungs, liver, bones, and lymph nodes	Capecitabine and cyclophosphamide	47 years	Right eye	Blurred vision in the right eye; one choroidal nodule with serous retinal detachment; 30 Gy right-eye radiation	47 years
3	24 years	Left	Mastectomy; lymph node dissection; breast implant; tamoxifen; leuprorelin	26 years	Bilateral lungs, lymph nodes, pleural dissemination, liver, brain	Paclitaxel; FEC regimen; epirubicin and cyclophosphamide; capecitabine and cyclophosphamide	27 years	Both eyes	Metamorphopsia in both eyes; one flat-shaped choroidal nodule in the right eye and two flat-shaped choroidal nodules in the left eye with serous retinal detachment; 30 Gy both-eye radiation	27 years
4	69 years	Right	Preoperative chemotherapy (FEC regimen and then gemcitabine and paclitaxel); mastectomy; lymph node dissection	71 years	Parasternal and mediastinal lymph nodes	Tegafur-gimeracil-oteracil for a year; 70 Gy mediastinal radiation; doxifluidine and cyclophosphamide	71 years	Right eye	Darkness in the right eye; two large choroidal nodules with extensive serous retinal detachment; 56 Gy right-eye radiation	72 years
5	37 years	Left	Mastectomy; sentinel lymph node biopsy and dissection; tamoxifen	43 years	Multiple bones, bilateral lungs, lymph nodes, and brain	Leuprorelin and anastrozole; doxifluidine and cyclophosphamide; toremifene; fulvestrant; letrozole; exemestane; AC regimen; docetaxel; capecitabine; CyberKnife radiotherapy to brain metastatic lesions; eribulin	50 years	Left eye	Shadow in left-eye vision; one choroidal nodule with serous retinal detachment; 30 Gy left-eye radiation	52 years

As regards the pathology of breast cancer (Table 2), immunostaining was not done in the earliest patient (Case 1) with breast cancer of invasive ductal carcinoma at that time of year 1987. Immunostaining of sections from the paraffin-embedded half-cut right eyeball preserved for 38 years revealed that the breast cancer cells in the choroidal metastatic lesion were positive for estrogen receptor and negative for progesterone receptor and HER2. The immunostaining for Ki-67 was not obtained due to the poor condition of the specimen. The exact pathological diagnosis of breast cancer was lost in the medical record of one patient (Case 2). Invasive ductal carcinoma in two patients (Case 3, Case 5) was positive for estrogen receptor, negative or positive for progesterone receptor, and negative for HER2. Invasive ductal carcinoma in the other patient (Case 4) was triple-negative for estrogen receptor, progesterone receptor, and HER2 with a high Ki-67 index. 

**Table 2:** Pathological features of breast lesions in five patients with choroidal metastasis from breast cancer Immunohistochemical results in Case 1 are derived from the restaining of the choroidal metastatic lesion. *: not stained due to the poor condition of the specimen HER2: human epidermal growth factor receptor 2

Case no.	Age at the diagnosis of breast cancer	Pathological diagnosis of breast lesion	Estrogen receptor	Progesterone receptor	HER2	Ki-67 index
1	32 years	Invasive ductal carcinoma	Positive	Negative	Negative	0%*
2	39 years	Not known	Not known	Not known	Not known	Not known
3	24 years	Invasive ductal carcinoma	Positive	Negative	Negative	<5%
4	69 years	Invasive ductal carcinoma	Negative	Negative	Negative	>30%
5	37 years	Invasive ductal carcinoma	Positive	Positive	Negative	<1%

Breast cancer is well-known to develop choroidal metastases in a large series of patients [9-12]. However, detailed descriptions of pathology, eye manifestations, and response to treatments in patients with choroidal breast cancer metastases have not been available in the literature. Thus, as a small series, we presented the eye manifestations of five patients with choroidal metastases from breast cancer in this study. We especially tried to document the response of choroidal metastatic lesions to chemotherapy and whole-eye radiation. In addition, pathological features were summarized to determine whether there would be a common feature in the pathology of breast cancer in patients with choroidal breast cancer metastases.

All five patients noticed eye symptoms which led to the diagnosis of choroidal metastases in the course of chemotherapy for systemic metastases. The choroidal metastases manifested as one or a few nodular or flat lesions with serous retinal detachment. In review of pathological findings in original lesions of breast cancer, cancer cells in invasive ductal carcinoma were positive for estrogen receptor and negative for HER2 in two patients, while they were triple-negative with a high Ki-67 index in one patient by immunostaining. In the earliest patient (Case 1), only a slide of the original breast lesion with hematoxylin-eosin staining was available from the previous hospital. Immunostaining of the choroidal metastatic lesion of the enucleated eye in 38-year preservation as a paraffin-embedded specimen revealed cancer cells positive for estrogen and negative for HER2. Immunostaining for Ki-67 was not confirmed probably due to the poor condition of the specimen. One needs to be cautious when interpreting the results of immunostaining since the original breast lesion and metastatic lesions may have a different pattern of immunostaining [13]. The record for pathological diagnosis was lost in another patient (Case 2). Altogether, the four patients with pathological confirmation in this series shared a HER2-negative feature, including a triple-negative feature, as a poor prognostic sign.

Fluorodeoxyglucose positron emission tomography is the standard for staging breast cancer [14-16] and has been done in four patients after the year 2006 when positron emission tomography became available in this area of Japan. Choroidal metastatic lesions showed the abnormal uptake of fluorodeoxyglucose as shown in Case 2 (Figure 2F). In the field of ophthalmology, tiny lesions of conjunctival extranodal marginal zone B-cell lymphoma of mucosa-associated lymphoid tissue (MALT) [17] as well as small intraocular lesions of choroidal malignant melanoma [2] and choroidal metastasis [5] can be detected by fluorodeoxyglucose positron emission tomography. 

Whole-eye radiation in four patients appeared to be effective in reducing the size of choroidal metastatic lesions, as reported in the literature [18-20]. In the latest patient (Case 5), the choroidal nodular lesion became smaller and flattened when she had four courses of doxorubicin and cyclophosphamide in three months which was then switched to six courses of docetaxel in six months. However, the choroidal lesion began to become larger at a faster rate one month after the end of the docetaxel course. Capecitabine was introduced but was ineffective in preventing the choroidal nodular lesion from growing rapidly. Whole-eye radiation to the left eye, which was chosen in dialogue with the patient and her daughter, appeared to be effective in stabilizing the rapid growth of the choroidal nodule. A palliative dose, totaling 30 Gy, was given in three patients, while a radical therapeutic dose, totaling 56 Gy, was given in one patient (Case 4) since this patient had an extremely large intraocular mass. 

## Conclusions

In the background of well-known choroidal metastases from breast cancer in a large series of patients, clinical eye manifestations in detail and their response to the treatment were described in a small series of five patients in a single referral-based institution. Pathologically, breast cancer in the diagnosis of invasive ductal carcinoma was positive for estrogen receptor and negative for HER2 in three patients, while it was triple-negative with a high Ki-67 index in one patient. The prognosis for life was poor in patients with breast cancer who developed choroidal metastases in the common background of a HER2-negative feature, including a triple-negative feature. Choroidal metastatic lesions showed a response to whole-eye radiation to improve the quality of vision at the end of life. In the era of continuing advances in the treatment options, patients with breast cancer metastases would survive longer in better health conditions and, thus, might be expected to experience choroidal metastases supposedly at a higher rate. The clinical description in this small series of patients is a major limitation in this study but would provide practical information to clinicians who encounter a patient with choroidal breast cancer metastasis on a rare occasion. The occurrence of choroidal metastasis as late as seven years in the most recent patient of this series is a reminder to keep patients on longer follow-up.
